# Supplementary material for: Content-rich biological network constructed by mining PubMed abstracts
Source: BMC Bioinformatics. 2004 Oct 8;5:147. doi: 10.1186/1471-2105-5-147 (PMC528731; doi:10.1186/1471-2105-5-147)
Supplement: Additional File 2 — The original results of the above study (non-essential files are deleted to keep the file size under the limit set by BMC bioinformatics). [file 1471-2105-5-147-S2.bz2 › chilibotAdditionalFile2/dip50/245ID8969169E801/html/IFNAR1_IFNAR2.html]

 


 **IFNAR1** and **IFNAR2** 
  
Found 94 abstracts in PubMed, retrieved 50.  
 

 What does Google say? 
 PDF only 
| .edu only 

---

**Interactive relationship** (e.g. stimulation, inhibition, etc)

**Neutral relationship**- The interaction of IFNs with their heterodimeric type I IFN receptor comprised of  **IFNAR1**  and  **IFNAR2**  is a first step to inducing biological actions.  Ref: 12542398 Biochem J, 2003
- Recombinant proteins corresponding to the cytoplasmic domains of the receptor subunits and the central core region of Stat2 were partially purified and used in affinity precipitation experiments to demonstrate that Stat2 binds more avidly to  **IFNaR2**  than to phosphorylated  **IFNaR1** .  Ref: 12220192 Biochemistry, 2002
- The ability to produce the IFN beta 1a  **IFNAR2**  and IFN beta 1a  **IFNAR1**   **IFNAR2**  complexes make them attractive candidates for X ray crystallography studies aimed at determining the molecular interactions between IFN beta 1a and its receptor.  Ref: 10493588 Protein Sci, 1999
- The mutants were analyzed for activity in vitro in antiviral and in antiproliferation assays, and for their ability to bind to the type I IFN  **ifnar1**   **ifnar2**  receptor on Daudi cells and to a soluble  **ifnar2**  fusion protein  **ifnar2**  Fc.  Ref: 10704203 Biochemistry, 2000
- Type I interferons bind to two cell surface receptors,  **ifnar1**  and  **ifnar2** , as the first step in the activation of several signal transduction pathways that elicit an anti viral state and an anti proliferative response.  Ref: 10984492 J Biol Chem, 2000
- Type I IFNs bind to a receptor composed of two transmembrane polypeptides,  **IFNAR1**  and  **IFNAR2** , which belong to the class II cytokine receptor family that also includes the cellular receptors for IFN gamma, interleukin 10 and coagulation protease factor VII tissue factor.  Ref: 11125298 Eur Cytokine Netw, 2000
- The interferon alpha receptor  [ **IFNAR1** ]  is composed of two subunits  **IFNaR1**  and  **IFNaR2** .  Ref: 12220192 Biochemistry, 2002
- Using anti receptor antibodies and mutant forms of beta interferon, three distinct modes of ligand interaction could be discerned i alpha interferon with  **ifnar1**  and  **ifnar2** .  Ref: 9737924 J Mol Biol, 1998
- The pleiotropic activity of type I interferons has been attributed to the specific interaction of IFN with the cell surface receptor components  **ifnar1**  and  **ifnar2** .  Ref: 11698684 Proc Natl Acad Sci U S A, 2001
- We conclude that the ternary complex forms by the sequential association of IFN beta 1a with  **IFNAR2** , followed by the association of  **IFNAR1**  with the preformed binary complex.  Ref: 10493588 Protein Sci, 1999
- Affinity precipitation experiments involving all three proteins Stat2, phosphorylated  **IFNaR1** , and  **IFNaR2**  indicated that the Stat2 receptor interactions are independent of one another.  Ref: 12220192 Biochemistry, 2002
- Hepatic expression of  **IFNAR1**  and  **IFNAR2**  were more frequent than PBMCs IFNAR  [ **IFNAR1** ]  3 27 85% vs 7 19 37%, P = 0.0021, IFNAR  [ **IFNAR1** ]  4 37 92% vs 19 29 66%, P = 0.0182.  Ref: 12141824 Dig Dis Sci, 2002
- The type I interferon receptor  [ **IFNAR2** ]  IFNAR  [ **IFNAR1** ]  is composed of two subunits, IFNAR 1  [ **IFNAR1** ]  and IFNAR  [ **IFNAR1** ]  2, encoding transmembrane polypeptides.  Ref: 10048764 J Interferon Cytokine Res, 1999
- The interferon alpha  [ **IFNAR1** ]  IFNalpha receptor consists of two subunits, the IFNalpha receptor 1  **IFNaR1**  and 2  **IFNaR2**  chains.  Ref: 11786546 J Biol Chem, 2002
- Stat2 bound  **IFNaR2**  K d = 45 nM approximately 6 fold stronger than it bound tyrosine 466 phosphorylated  **IFNaR1**  K d = 245 nM.  Ref: 12220192 Biochemistry, 2002
- The class II cytokine receptor CIICR genes Il10r2 and  **Ifnar1**  are localized on mouse Chr 16 in a cluster that also contains the CIICR genes  **Ifnar2**  and Ifngr2.  Ref: 12584606 Mamm Genome, 2003

**Non-interactive relationship** (e.g. studied together, co-existance, homology, etc.)

- Expression of interferon receptor subunits,  **IFNAR1**  and  **IFNAR2** , in the ovine uterus.  Ref: 12193393 Biol Reprod, 2002
- Gel filtration of a mixture of  **IFNAR1**  and the IFN beta 1a  **IFNAR2**  complex indicated that the three proteins formed a stable ternary complex.  Ref: 10493588 Protein Sci, 1999
- The soluble murine type I interferon receptor  [ **IFNAR2** ]  Ifnar  [ **IFNAR1** ]  2 is present in serum, is independently regulated, and has both agonistic and antagonistic properties.  Ref: 11154225 Blood, 2001
- To assess whether hepatitis C virus infection HCV affects type I interferon IFN receptor expression, the expression levels of two subunits of this receptor,  **IFNAR1**  and  **IFNAR2** , in peripheral blood mononuclear cells PBMCs were determined by flow cytometric assay in 68 anti HCV antibody positive patients 47 positive for HCV RNA and 21 negative for HCV RNA and 14 healthy controls.  Ref: 12787799 Hepatol Res< MedlineTA>Hepatol Res, 2001
- We conclude that alpha and beta interferons signal differently through their receptors because the two ligand subtypes interact with the receptor subunits ifnar 1  [ **IFNAR1** ]  and  **ifnar2**  in entirely different ways.  Ref: 9737924 J Mol Biol, 1998
- Immunostaining of  **IFNAR1**  and  **IFNAR2**  was observed in the cell membrane and cytoplasm of hepatocytes in all liver specimens.  Ref: 12699848 Hepatol Res< MedlineTA>Hepatol Res, 1998
- In addition, we sought for genetic polymorphism in the promoter region of the interferon alpha  [ **IFNAR1** ]  beta receptor 2 gene  **IFNAR2** , and found single nucleotide polymorphisms SNPs at 134 and 75.  Ref: 12697242 Hepatol Res< MedlineTA>Hepatol Res, 1998
- Mapping human interferon alpha  [ **IFNAR1** ]  IFN alpha 2 binding determinants of the type I interferon receptor  [ **IFNAR2** ]  subunit IFNAR 1  [ **IFNAR1** ]  with human bovine IFNAR 1  [ **IFNAR1** ]  chimeras.  Ref: 9737881 Biochemistry, 1998
- The extracellular portions of the chains that comprise the human type I interferon receptor  [ **IFNAR2** ] ,  **IFNAR1**  and  **IFNAR2** , have been expressed and purified as recombinant soluble His tagged proteins, and their interactions with each other and with human interferon beta 1a IFN beta 1a were studied by gel filtration and by cross linking.  Ref: 10493588 Protein Sci, 1999
- To determine whether phosphorylation of tyrosine residues in the IFN receptor is required for activation of the PI 3 kinase, we performed studies using mouse L929 fibroblasts transfected with mutated human  **IFNAR1**  and or  **IFNAR2**  subunits of the Type I IFN receptor, lacking tyrosine phosphorylation sites.  Ref: 10733921 Biochem Biophys Res Commun, 2000
- Alternative splicing leads to the expression of multiple isoforms of the subunits  **IFNAR1**  and  **IFNAR2**  of the type I IFN receptor.  Ref: 11344274 Proc Natl Acad Sci U S A, 2001
- Type I interferons are cytokines which activate an anti viral response by binding to two specific cell surface receptors,  **ifnar1**  and  **ifnar2** .  Ref: 10339405 J Mol Biol, 1999
- The potent antiviral and antiproliferative activities of human type I interferons IFNs are mediated by a single receptor comprising two subunits,  **IFNAR1**  and  **IFNAR2** .  Ref: 12842042 Structure Camb , 2003
- For this report, we tested the pharmacogenomic effects of eight polymorphisms in the interferon receptor genes  **IFNAR1**  and  **IFNAR2**  in a group of 147 patients undergoing open label IFNbeta therapy.  Ref: 12618863 Genes Immun, 2003
- These studies demonstrate the heavy concentration of  **IFNAR1**  and  **IFNAR2**  in cells of the LE and superficial GE, which appear to be the main targets for IFN tau.  Ref: 12193393 Biol Reprod, 2002
- In multiple regression analysis, the presence of HCV RNA in serum was independently associated with the expression of both  **IFNAR1**  and  **IFNAR2**  in PBMCs P=0.007 for  **IFNAR1**  and P=0.003 for  **IFNAR2** .  Ref: 12787799 Hepatol Res< MedlineTA>Hepatol Res, 2002
- Analysis of ternary complex formation using various molar excesses of  **IFNAR1**  and the IFN beta 1a  **IFNAR2**  complex indicated that the ternary complex had a 1 1 1 stoichiometry, and reducing SDS PAGE of the ternary complex treated with DSG indicated that the major cross linked species had an apparent Mr consistent with the sum of its three individual components.  Ref: 10493588 Protein Sci, 1999
- By gel filtration, no stable binary complexes between IFN beta 1a and  **IFNAR1** , or between  **IFNAR1**  and  **IFNAR2**  were detected.  Ref: 10493588 Protein Sci, 1999
- Liver biopsy specimens from 41 chronic hepatitis C patients subsequently treated with IFN were immunostained with  **IFNAR1**  and  **IFNAR2**  antibodies, and also analyzed for both receptor subunit mRNA levels, using competitive polymerase chain reaction.  Ref: 12699848 Hepatol Res< MedlineTA>Hepatol Res, 1999
- Hepatic expression of  **IFNAR1**  r=0.45, P=0.012 or  **IFNAR2**  mRNA r=0.46, P=0.009 was weakly correlated with their protein expression in hepatocytes.  Ref: 12699848 Hepatol Res< MedlineTA>Hepatol Res, 1999
- In the type I interferon receptor  [ **IFNAR2** ] , Tyk2 associates with the  **IFNAR1**  receptor subunit and positively influences ligand binding to the receptor complex.  Ref: 12554654 EMBO J, 2003
- We report that bone marrow derived macrophages BMM lacking a component of the type I interferon receptor  [ **IFNAR2** ]  IFNAR 1  [ **IFNAR1** ]  do not express cyclin D2 mRNA or protein in response to LPS stimulation 0.01 1 microg ml for 7 30 h.  Ref: 10805369 J Interferon Cytokine Res, 2000
- Here we describe two transcripts representing extracellular forms of ovine  **IFNAR1**  and show that soluble extracellular forms of both  **IFNAR2**  and  **IFNAR1** , prepared in recombinant form in Escherichia coli, have antiviral AV activity in the absence of IFN.  Ref: 11344274 Proc Natl Acad Sci U S A, 2001
- The percentages of  **IFNAR1**  and  **IFNAR2**  expressing cells were significantly higher in HCV RNA positive patients than in HCV RNA negative patients or healthy controls.  Ref: 12787799 Hepatol Res< MedlineTA>Hepatol Res, 2001
- The precise role of the different subunits alpha  **IFNAR1**  and betaL  **IFNAR2**  of the type I interferon receptor  [ **IFNAR2** ]  IFN R  [ **IFNAR2** ]  in the activation of signal transducer and activator of transcription Stat 1, Stat2, and Stat3 has not yet been established.  Ref: 9933596 J Biol Chem, 1999
- This receptor is comprised of subunits  **IFNAR1**  and  **IFNAR2** , the latter exhibiting nanomolar affinity for IFNs.  Ref: 11888273 Biochemistry, 2002
- The labeling indexes of  **IFNAR1**  136.7 94.1 vs. 77.4 76.8, P=0.032 and  **IFNAR2**  153.1 80.2 vs. 87.2 75.5, P=0.011 in liver specimens were significantly higher in sustained virologic responders n=17 than in non sustained virologic responders n=24, while mRNA levels of either receptor subunit did not differ between response groups.  Ref: 12699848 Hepatol Res< MedlineTA>Hepatol Res, 2002
- To explore the function of the Stat2  **IFNaR2**  interaction and its possible relationship to the SH2 dependent docking of Stat2 to phosphorylated  **IFNaR1** , the affinity of Stat2 for each of the receptor subunits was determined.  Ref: 12220192 Biochemistry, 2002
- In situ hybridization and immunohistochemistry on sections from pregnant and nonpregnant ovine uteri at Days 14 and 15 after estrus and mating showed that both  **IFNAR1**  and  **IFNAR2**  mRNA and protein were strongly expressed in endometrial luminal epithelium LE, superficial glandular epithelium GE, and stromal cells, within but not outside caruncles.  Ref: 12193393 Biol Reprod, 2002
- ii beta interferon with  **ifnar1**  and  **ifnar2** .  Ref: 9737924 J Mol Biol, 1998
- The focus of this study is to determine both mRNA and protein expression levels of the type I interferon IFN receptor subunits, IFNalpha receptor  **IFNAR1**  and IFNalpha beta receptor  **IFNAR2** , in the liver, and to assess which quantification method is most useful in predicting response to IFN in chronic hepatitis C patients.  Ref: 12699848 Hepatol Res< MedlineTA>Hepatol Res, 1998
- Their receptors,  **IFNAR1**  and  **IFNAR2** , belong to the class II cytokine receptor family for a helical cytokines.  Ref: 11544103 Cytokine Growth Factor Rev, 2001
- METHODS HBc specific proliferative responses and cytokine release of blood mononuclear cells PBMC were studied in patients with AHB or CHB, as well as responders and non responders to interferon alpha  [ **IFNAR1** ]  treatment IFN R  [ **IFNAR2** ] , IFN NR, by 3H thymidine uptake, enzyme linked immunosorbent assay ELISA and Elispot assay and were compared to peptide HBc18 27 specific CTL precursor frequencies among CD8 T cells derived from HLA A2 patients.  Ref: 9869395 Liver, 1998
- Structure function study of the extracellular domain of the human type I interferon receptor  [ **IFNAR2** ]  IFNAR 1  [ **IFNAR1** ]  subunit.  Ref: 10841076 J Interferon Cytokine Res, 2000
- To assess whether peripheral blood mononuclear cells PBMCs can be substituted for liver tissue in monitoring the hepatic expression of interferon receptors  **IFNAR1**  and or  **IFNAR2**  in chronic hepatitis C, we quantified the mRNA of both subunits, using the competitive polymerase chain reaction, in the liver specimens from 37 chronic hepatitis C patients and in PBMCs from 29 of 37 patients.  Ref: 12141824 Dig Dis Sci, 2002
- This cluster includes both components of the interferon alpha  [ **IFNAR1** ]  beta receptor  **IFNAR1**  and  **IFNAR2**  and the second components of the interferon gamma receptor IFNGR2 and of the IL 10 receptor IL10R2.  Ref: 10077530 Genome Res, 1999
- We report here the complete gene content of this GART cytokine receptor gene cluster and the use of comparative genomic analysis to identify chicken  **IFNAR1** ,  **IFNAR2** , and IL10R2.  Ref: 10077530 Genome Res, 1999
- Type I interferons IFN exert pleiotropic activities through binding to two cell surface receptors,  **ifnar1**  and  **ifnar2** .  Ref: 10556041 J Mol Biol, 1999
- We constructed chimeric receptors wherein the extracellular domain of the erythropoietin receptor EpoR was fused to the transmembrane and intracellular domains of the interferon IFN type I receptor subunits,  **IFNaR1**  or  **IFNaR2**  2.  Ref: 10574956 J Biol Chem, 1999
- The type I interferon family includes 13 alpha, one omega and one beta subtypes recognized by a complex containing the receptor subunits  **ifnar1**  and  **ifnar2**  and their associated Janus tyrosine kinases, Tyk2 and Jak1.  Ref: 9737924 J Mol Biol, 1998
- The data suggest that AV activity is conferred by the ability of soluble R2E to associate with the transfected  **IFNAR2**  subunit rather than resident murine  **IFNAR1** .  Ref: 11344274 Proc Natl Acad Sci U S A, 2001
